# Le traitement conservateur de la pyélonéphrite emphysémateuse chez les patients diabétiques: à propos de cinq observations

**DOI:** 10.11604/pamj.2016.25.151.6976

**Published:** 2016-11-14

**Authors:** Aziz El Majdoub, Abdelhak Khallouk, Moulay Hassan Farih

**Affiliations:** 1 Service d’Urologie, Centre Hospitalier Universitaire Hassan II, Fès, Maroc

**Keywords:** Pyélonéphrite emphysémateuse, diabète, traitement conservateur, Emphysematous pyelonephritis, diabetes, conservative treatment

## Abstract

La pyélonéphrite aigue emphysémateuse (PNE) est une forme grave d'infection rénale. Elle est grevée d'une lourde mortalité en dehors d'un traitement rapide et efficace. Notre travail vise à préciser les caractéristiques cliniques et paracliniques ainsi que les particularités de la prise en charge thérapeutique de cette pathologie et nous mettons l'accent sur la possibilité d'un traitement conservateur chez le diabétique. Nous avons analysé les dossiers médicaux des patients traités pour PNE au service d'urologie du CHU Hassan II de Fès entre Janvier 2004 et janvier 2010. Pour chaque dossier, nous avons précisé les caractéristiques cliniques, paracliniques et thérapeutiques ainsi que l'évolution après traitement. Il s'agissait de 5 patientes dont l'âge moyen était de 45,6 ans. Elles étaient toutes diabétiques. Une obstruction d'origine lithiasique des voies excrétrices supérieures a été retrouvée chez 3 patientes (60%). Le diagnostic avait été porté par l'intermédiaire de la tomodensitométrie (TDM) abdominale. Toutes les patientes avaient bénéficié des mesures de réanimation associant notamment une antibiothérapie et insulinothérapie. Le traitement chirurgical était conservateur dans tous les cas. En effet, un drainage chirurgical des collections péri rénales était réalisé dans deux cas, un drainage rénal percutané dans un cas et un drainage par une sonde urétérale double JJ dans 2 cas. L'évolution clinique et radiologique était excellente avec conservation rénale chez toutes les patientes. La pyélonéphrite emphysémateuse est une complication rare et grave, en particulier chez un patient diabétique. Le diagnostic positif sur la TDM. Le traitement chirurgical doit être conservateur au maximum, en dehors des formes graves, surtout chez le patient diabétique qui est un risque potentiel d'insuffisance rénale chronique.

## Abstract (en)

Acute emphysematous pyelonephritis (EPN) is a severe kidney infection. Early and effective treatment is essential to reduce high mortality rates due to this disease. Our study aims to highlight the clinical and paraclinical features as well as the peculiarities of therapeutic management of this disease and focus on the possibility of conservative treatment in diabetic patients. We analyzed the medical records of patients with EPN treated in the Department of Urology at the Hassan II University Hospital, Fez between January 2004 and January 2010. For each medical record we described clinical, paraclinical and therapeutic features as well as patient’s evolution after treatment. We here report the case of 5 female patients whose average age was 45,6 years. All patients were diabetic. Lithiasic obstruction of the upper urinary tract was found in 3 patients (60%). The diagnosis was made by means of abdominal CT scan. All patients underwent resuscitation measures including antibiotic and insulin therapy. Conservative surgical procedure was performed in all cases. Indeed, surgical drainage of perirenal collections was performed in two cases, percutaneous renal drainage in one case and drainage using double-J ureteral catheter in 2 cases. Clinical and radiological evolution was excellent with renal preservation in all patients Emphysematous pyelonephritis is a rare and serious complication, especially in diabetic patients. Diagnosis is based on CT scan. Surgical treatment should be conservative in most cases, apart from severe forms, especially in diabetic patients who have potential risk of chronic renal failure.

## Introduction

La pyélonéphrite emphysémateuse (PNE) est une forme sévère et nécrosante de la pyélonéphrite bactérienne aiguë. Elle est caractérisée par la présence de gaz au sein du parenchyme rénal, des cavités excrétrices et/ou des espaces périrénaux. Elle est habituellement rencontrée chez les patients diabétiques notamment de sexe féminin. Sa présentation clinique initiale peut être trompeuse et son diagnostic repose essentiellement sur la tomodensitométrie qui permet en plus d'évaluer son pronostic et de suivre son évolution. Elle est grevée d'une lourde mortalité en dehors d'une prise en charge médicochirurgicale rapide. Nous mettons l'accent sur la possibilité d'un traitement conservateur qui permet aux patients de conserver un capital néphronique précieux notamment chez le diabétique.

## Méthodes

Nous avons étudié les dossiers médicaux des patients traités pour pyélonéphrite aigue emphysémateuse PNE au service d'urologie du CHU Hassan II de Fès entre 2004 et 2009. Pour chaque dossier, Nous avons recueilli rétrospectivement les données épidémiologiques, cliniques, biologiques, radiologiques, thérapeutiques et évolutives.

## Résultats

Nous avions identifié 5 cas de pyélonéphrite emphysémateuse. Tous nos patients étaient de sexe féminin et diabétiques connues. L'âge moyen était de 45,6 ans (37-56 ans). Une obstruction des voies excrétrices supérieure avait été retrouvée chez 3 patientes (60%). Il s'agissait d'une lithiase rénale pyélique dans un cas et d'une lithiase urétérale chez les deux autres patientes. L'atteinte rénale était localisée du côté gauche chez 3 patientes (60%). Le délai moyen de consultation était de 5,8 jours (5 - 7 jours) après le début de la symptomatologie. Les lombalgies fébriles avec altération de l'état général étaient le principal motif de consultation chez toutes nos patientes. Le tableau clinique était celui d'une pyélonéphrite aiguë typique commune dans 2 cas. Chez une patiente, l'examen avait retrouvé une tuméfaction inflammatoire lombaire avec une défense lombaire et deux patientes présentaient d'emblée des troubles de conscience en rapport avec un état d'acidocétose diabétique. Sur le plan biologique, La numération sanguine avait objectivé une hyperleucocytose chez toutes les malades, allant de 13000 à 30000 éléments/mm^3^, avec une anémie hypochrome microcytaire dans 3 cas. Le dosage de la CRP était supérieur à 100mg/l chez toutes nos patientes. Une hyperglycémie était noté dans tous les cas avec des valeurs extrêmes de l'ordre de 5,3 g/l. seules deux patientes présentaient une insuffisance rénale aigue fonctionnelle en rapport avec le sepcis qui avait régressé après les mesures de réanimation. L'examen urinaire par bandelette réactive avait permis de diagnostiquer une décompensation acidocétosique du diabète chez toutes nos patientes et l'examen cytobactériologique des urines était positif dans tous les cas, avec un Escherichia Coli isolé dans 4 cas (80%) et un proteus mirabilis dans un cas. L'arbre urinaire sans préparation (AUSP) avait montré des clartés gazeuses sur l'aire rénale dans deux cas et une lithiase calcique se projetant sur l'arbre urinaire dans trois cas.L'échographie avait été réalisée chez toutes nos patientes et avait objectivé une interposition de gaz dans 2 cas et des échos de réverbérations au sein du parenchyme rénal dans 3 cas. Le diagnostic avait été porté par l'intermédiaire d'un examen tomodensitométrique dans tous les cas. Il avait montré la présence de gaz dans le parenchyme rénale et les voies excrétrices dans 3 cas et une diffusion de l'air dans les espaces rétropéritonéaux dans deux cas. Par ailleurs la TDM avait permis de faire le diagnostic de lithiase rénale et urétérale chez les trois patientes qui avaient une obstruction des voies urinaires excrétrices. Toutes les patientes avaient bénéficié de mesures de réanimation pour rétablir l'équilibre hydroélectrolytique, d'une insulinothérapie et d'une bi-antibiothérapie comportant une céphalosporine de troisième génération et un aminoside. Un drainage chirurgical des collections péri-rénales avait été réalisé dans deux cas. Chez trois patientes, un drainage des voies excrétrices était nécessaire par le moyen d'une nephrostomie percutanée dans un cas et par une sonde urétérale double J dans 2 cas. L'évolution clinique et radiologique était excellente avec conservation rénale chez toutes nos patientes.

## Discussion

La pyélonéphrite emphysémateuse est une infection nécrotique du rein caractérisée par la présence de gaz au sein du parenchyme rénal, des cavités excrétrices ou des espaces péri-rénaux. L'âge moyen de survenue est de 53 ans, avec une prédominance du sexe féminin [1–4]. L'atteinte rénale prédomine du coté gauche (60%). Les formes bilatérales sont rares (5-20%) et sont particulièrement graves (mortalité 20 fois supérieure) [5]. Le facteur étiologique le plus fréquemment retrouvée est le diabète, a fortiori mal équilibré [4, 5]. Cela s'explique par l'hyperglycémie chronique qui favorise la microangiopathie, les anomalies anatomiques et fonctionnelles du tractus urinaire, et les anomalies de l'immunité antibactérienne. Egalement, la neuropathie diabétique retarde le diagnostic en réduisant la symptomatologie douloureuse et favorise la survenue de formes graves [5]. Le deuxième facteur étiologique est l'existence d'un obstacle sur les voies urinaires (20 à 41% des cas) [6, 7]. La physiopathologie de la PNE est encore discutée. La principale hypothèse est celle de la fermentation intrarénale du glucose en présence de germes gram négatif facultativement anaérobies dans un environnement tissulaire favorable (un taux de glucose intratissulaire élevé, une perfusion tissulaire défectueuse et une réponse immunitaire altérée) [3, 5, 8]. Le gaz se forme d'abord autour de la papille où la vascularisation est pauvre puis il passe dans le pyélon et fuse le long des pyramides et dans l'espace périnéphrétique. Le diagnostic est souvent retardé entre 1 à 3 semaines après le début des symptômes [5]. Le tableau clinique comprend des signes d'une pyélonéphrite grave, avec de la fièvre et des frissons. Des signes en rapport avec un état septicémique ou la décompensation d'un diabète sont souvent associés. Un contact lombaire n'est retrouvé que dans 50% des cas et la palpation d'une crépitation de la fosse lombaire est évocatrice mais rarement retrouvée [9]. Les examens biologiques permettent de confirmer le sepsis et de rechercher une décompensation du diabète. Ils permettent également de rechercher des facteurs de gravité sous forme d'une dysfonction viscérale (insuffisance rénale, insuffisance hépatique) ou d'une coagulopathie de consommation (thrombopénie, augmentation du temps de céphaline activée et baisse du taux de prothrombine) [5, 10, 11]. L'enquête bactériologique repose essentiellement sur l'examen cytobactériologique des urines. L'analyse des urines met en évidence une leucocyturie et une hématurie dont l'importance reflète le degré de nécrose ou de destruction du rein par le processus infectieux. La culture est positive une fois sur deux et les micro-organismes identifiés sont représentées essentiellement par les bacilles à Gram négatif, surtout E. coli (80% dans notre série) et Klebsiella pneumoniae et Proteus mirabilis. Les germes anaérobies restent exceptionnels et certains cas de PNE imputés à des levures ont été décris [10–12]. Les hémocultures n'ont de valeur que si elles sont positives et qu'elles isolent le même germe que celui retrouvé dans les urines. L'exploration radiologique constitue la clé du diagnostic positif de la PNE. L'arbre urinaire sans préparation peut révéler un emphysème rénal (65 %) ou un rétro-pneumopéritoine et permet de détecter d'éventuels obstacles lithiasiques radio-opaques. L'échographie rénale est d'interprétation difficile. Elle peut montrer des zones hyperéchogènes avec atténuation postérieure et réverbération correspondant aux bulles de gaz, mais elle ne permet pas un bilan d'extension précis de la maladie. Elle recherche aussi une obstruction de la voie excrétrice et peut orienter vers la nature de l'obstacle [11–13]. La tomodensitométrie est l'examen de référence pour le diagnostic et le suivi de la pyélonéphrite emphysémateuse [7]. Elle est sensible (100%) pour détecter la présence d'un épanchement gazeux sous forme de densité fortement négative et apprécier la destruction parenchymateuse. Elle permet également d'étudier les espaces périrénaux et précise ainsi l'extension des lésions. L'injection de produit de contraste n'est pas indispensable, d'autant qu'elle fait courir le risque d'insuffisance rénale aiguë chez ces patients [5]. Wan et al distinguent deux types de PNE selon la présentation scannographique avec une valeur pronostique et un impact thérapeutique [14]: le type 1: destruction parenchymateuse et absence de collection ou existence de gaz intra- et/ou périrénal sous forme de striations; le type 2 est caractérisé par la présence de gaz intra- et/ou périrénal sous forme de bulles avec en plus présence de gaz dans le système collecteur ou d'une collection intra- ou périrénale.

Huang et Tseng ont établi une autre classification scannographique ayant un impact thérapeutique [8]. Elle classe les PNE en 4 stades: stade 1: gaz dans les voies excrétrices seulement; stade 2: gaz dans le parenchyme rénal sans extension dans l'espace extrarénal; stade 3A: extension du gaz ou abcès de la loge rénale; stade 3B: extension du gaz ou abcès au delà du fascia de Gérota; stade 4: pyélonéphrite emphysémateuse bilatérale ou sur rein unique.

**Figure 1:**
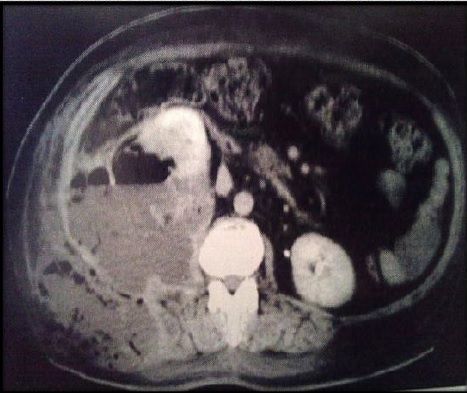
TDM montrant une PNE du rein droit avec une collection périrénale s’étendant aux parties molles de la région lombaire (stade 3 de Huang et Tseng)

**Figure 2:**
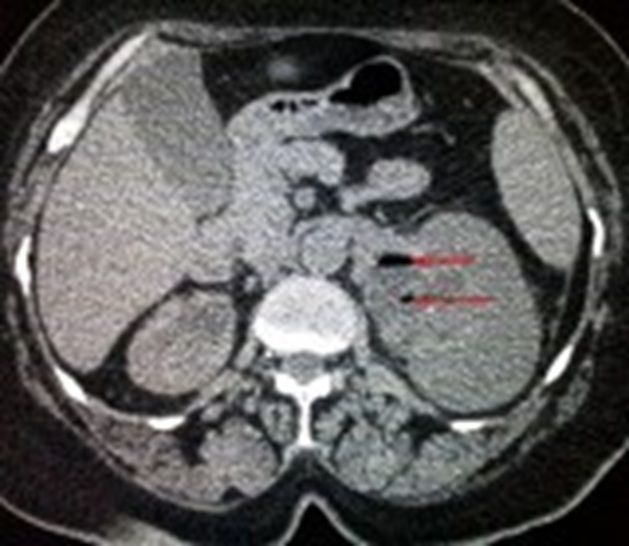
TDM montrant une PNE du rein gauche stade 2 de Huang et Tseng

**Figure 3:**
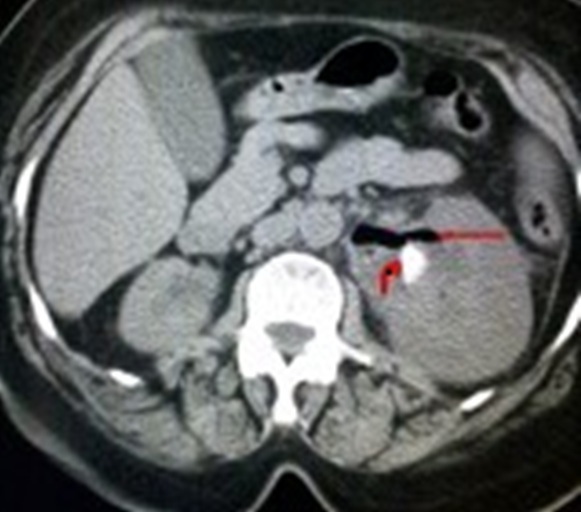
Coupe scannographique sans injection montrant une PNE gauche sur lithiase pyélique (stade 1)

Deux parmi nos patientes avaient été classées stade 3B (PNE unilatérale avec présence de collections péri rénales s'étendant au delà du fascia de Gérota et atteignant même les parties molles lombaires chez une patiente) (Figure 1), deux patientes avaient présenté un stade 2 (Figure 2) et une patiente était classée stade 1 (Figure 3). Nous avons constatés que les patientes qui avaient une obstruction, avaient présenté des tableaux cliniques plus sévères et nous pensons que l'obstruction est un facteur de gravité et doit être signalé dans la classification. Le pronostic des PNE est globalement sévère avec un taux de mortalité de 19%, toutes thérapeutiques confondues [5]. En effet, ce pronostic est d'autant plus fâcheux, selon Wan et al, qu'il s'agit d'un type 1, qu'il existe une insuffisance rénale (créatininémie supérieure à 120 µmol/l), une thrombopénie (inférieure à 60.000 éléments/mm^3^) ou une hématurie [14]. Le pronostic de la fonction rénale à long terme dépend de degré de destruction parenchymateuse et de l'existence d'une néphropathie associée. D'où l'intérêt du traitement conservateur dans la mesure du possible, particulièrement chez le sujet diabétique. La prise en charge de la pyélonéphrite emphysémateuse est une urgence médico-chirurgicale. Elle repose sur l'association d'une réanimation vigoureuse, d'une antibiothérapie probabiliste active sur les bacilles à gram négatif et d'un geste de drainage chirurgical percutané et/ou endourologique. Le traitement symptomatique des troubles hémodynamiques, hydro-électrolytiques et des dysfonctions d'organes ainsi que le contrôle de l'hyperglycémie sont des mesures non spécifiques mais indispensables. Ils doivent se faire en service de soins intensifs. L'antibiothérapie probabiliste initiale associe une céphalosporine de troisième génération ou l'imipénème à une fluoroquinolone ou un aminoside. Les fluoroquinolones ont l'avantage d'une excellente diffusion tissulaire et d'une faible toxicité. Cette antibiothérapie initiale sera adaptée secondairement en fonction des résultats bactériologiques et de l'efficacité clinique. Les moyens chirurgicaux de la pyélonéphrite emphysémateuse comportent: Le drainage des cavités excrétrices par une sonde de néphrostomie percutanée ou par une sonde urétérale simple ou en double J: ce type de drainage est indiqué dans les formes localisées à la voie excrétrice (stade 1 de Huang) ou en cas d'obstruction [12, 13, 15, 16]. Le drainage percutané de la loge rénale et des espaces périrénaux représente, depuis sa première description par Hudson en 1986 [17], de plus en plus le gold standard thérapeutique dans les stades 3 et 4. En effet, le drainage percutané doit constituer le premier volet d'une démarche thérapeutique gradué pouvant comporter par la suite une néphrectomie en présence de facteurs de mauvais pronostic. La durée moyenne du drainage est de 1-5 semaines. Une TDM de contrôle sera réalisée entre le quatrième et le septième jour à la recherche de nouvelles éventuelles collections qui devraient être drainées par d'autres cathéters.

Le drainage chirurgical de la loge rénale et des espaces périrénaux dans les stades 3 et 4 en cas de collections étendues et cloisonnées. Ce type de drainage était réalisé chez deux de nos patientes qui avaient des collections cloisonnées s'étendant à la région lombaire.

La néphrectomie: elle était considérée autrefois par la plupart des équipes comme le traitement de référence de la pyélonéphrite emphysémateuse. Actuellement, elle doit être indiquée en deuxième intention après échec du traitement conservateur ou exceptionnellement en première intention de sauvetage en cas de formes extensives avec plusieurs dysfonctions d'organes [5, 18].

## Conclusion

La pyélonéphrite emphysémateuse est une infection rénale grave mettant en jeu le pronostic vital. Les patientes diabétiques sont particulièrement exposées à cette infection. La tomodensitométrie est l'examen de référence pour le diagnostic positif et le suivi. Le traitement conservateur doit être toujours tenté et la néphrectomie de première intention doit être réservé aux situations suivantes: formes graves extensives avec dysfonctions d'organes (foie, rein); formes avec rein non fonctionnel et détruit par le processus infectieux.

### Etat des connaissances actuelle sur le sujet

- La pyélonéphrite emphysémateuse est une complication rare et grave, en particulier chez un patient diabétique;
- Elle est grevée d’une lourde mortalité en dehors d’un traitement rapide et efficace;
- Le traitement chirurgical doit être conservateur au maximum, en dehors des formes graves, surtout chez le patient diabétique qui est un risque potentiel d’insuffisance rénale chronique.

### Contribution de notre étude à la connaissance

- Apporter notre expérience à travers cette série de cas dans la prise en charge de la pyélonéphrite emphysémateuse;
- Préciser les caractéristiques cliniques et paracliniques ainsi que les particularités de la prise en charge thérapeutique de cette pathologie;
- Le traitement chirurgical doit être conservateur au maximum, en dehors des formes graves, surtout chez le patient diabétique qui est un risque potentiel d’insuffisance rénale chronique.
